# An evaluation of in-office flexible fiber-optic biopsies for laryngopharyngeal lesions

**DOI:** 10.1186/s40463-018-0275-x

**Published:** 2018-05-09

**Authors:** Francisco Lee, Kristine A. Smith, Shamir Chandarana, T. Wayne Matthews, J. Douglas Bosch, Steven C. Nakoneshny, Joseph C. Dort

**Affiliations:** 1 Section of Otolaryngology – Head and Neck Surgery, Department of Surgery, Cumming School of Medicine, University of Calgary, HRIC 2A02, 3280 Hospital Dr NW, Calgary, AB T2N 4Z6 Canada; 2 Ohlson Research Initiative, Arnie Charbonneau Cancer Institute, Cumming School of Medicine, University of Calgary, Calgary, Alberta Canada

**Keywords:** Endoscopy, In-office biopsy, Larynx, Pharynx, Lesion

## Abstract

**Background:**

Operative endoscopy and flexible fiber-optic in-office tissue biopsy are common techniques to assess suspicious laryngopharyngeal lesions.

**Methods:**

The primary outcome was the delay to the initiation of treatment. Secondary outcomes were delay to biopsy, histopathological diagnosis, and assessment at a multidisciplinary oncology clinic. A retrospective analysis was performed to assess the relative delays between these approaches to biopsy of laryngopharyngeal lesions.

**Results:**

There were 114 patients in the study cohort; 44 in-office and 70 operative endoscopic biopsies). The mean delay from consultation to biopsy was 17.4 days for the operative endoscopy group and 1.3 days for the in-office group. The mean delay from initial otolaryngology consultation to initiation of treatment was 51.7 days and 44.6 days for the operative endoscopy and in-office groups, respectively.

**Conclusion:**

In-office biopsy reduced the time from initial consultation to biopsy. The temporal gains via in-office biopsy did not translate into faster access to treatment. This outcome highlights the opportunity to improve access to treatment for patients with early diagnosis.

## Background

Laryngopharyngeal lesions encompass a wide range of disease processes that includes benign lesions, local manifestations of systemic disease, inflammatory disorders and primary malignancies. In the United States, an estimated 61,700 new cases of oral cavity, pharynx, and larynx cancer will arise in 2016 and an estimated 13,190 deaths will occur from these cancers combined. [1] Given that early identification and treatment of head and neck cancers improves prognosis, [2–5] timely evaluation and diagnosis of suspicious lesions is important.

The larynx and lower pharynx are potentially challenging areas to assess and operative endoscopy is often necessary for thorough evaluation and biopsy of suspicious lesions. Operative endoscopy requires operating room (OR) time and general anesthesia and uses costly healthcare resources such as operating rooms and OR personnel. Complications arising from rigid instrumentation and general anesthesia, although uncommon, do occur, especially in high-risk patients with comorbid conditions.

One alternative, flexible fiber-optic nasopharyngoscopy, offers excellent visualization of the aerodigestive tract in an awake patient with low risk of complications. Newer technologies such as high-definition distal-chip nasopharyngoscopes provide excellent image quality, and side channels facilitate tissue biopsy with diagnostic accuracy comparable to that of operative endoscopy [6]. In a study by Castillo Farias et al. (2014), in-office biopsy of suspicious laryngeal lesions under fiber optic visualization offered a specificity of 81% and sensitivity of 100% compared to direct laryngoscopy [7]. Moreover, by performing an in-office biopsy as a first-line diagnostic step, and avoiding anesthetic and OR expenses, the authors reported a cost savings of 80% when compared to an operative biopsy for all patients presenting with suspicious lesions.

In addition to cost savings, it is important to understand the impact on time to diagnosis of different diagnostic approaches. The time differences between in-office and operative approaches to the biopsy of suspicious laryngopharyngeal lesions remains relatively unknown. Furthermore, little is known about the impact of time to diagnosis on overall time to initiation of treatment. We therefore undertook a study to measure the differences in time to diagnosis and time to initiation of treatment in patients with laryngopharyngeal lesions who were undergoing operative endoscopy compared to a patient cohort undergoing in-office biopsy. Our hypothesis was that in-office biopsy would result in faster diagnosis and faster access to treatment.

## Methods

### Study design

We performed a retrospective case-control study comprising patients referred to a tertiary Head and Neck Oncology and Laryngology Clinic from January 1st, 2010 to December 31st, 2015. Cases were defined as patients undergoing in-office biopsies and the controls were defined as patients undergoing operative endoscopy requiring general anesthesia. This study was reviewed and approved by the Health Research Ethics Board of Alberta – Cancer Committee.

### Patients

**Fig. 1:**
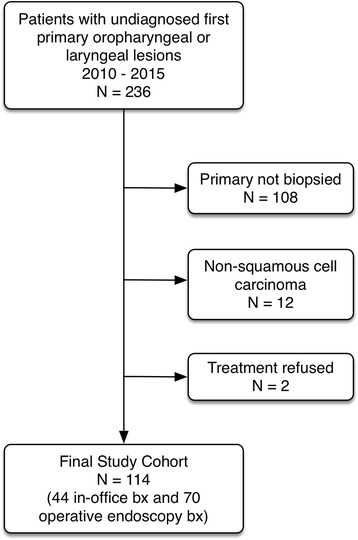
Diagram showing composition of final study cohort

The study focused on patients presenting with oropharyngeal or laryngeal lesions requiring biopsy. These subsites were chosen because conventional access and biopsy of these lesions traditionally requires operative endoscopy. Patients older than 18 years of age with lesions visible on office endoscopy were eligible for inclusion. In-office biopsy patients were identified by review of billing codes and laryngology clinic records during the time period of interest and charts of potentially eligible patients were reviewed. Operative endoscopy patients were identified by querying a relational database (Otobase®, Seattle, WA) used to prospectively track all patients treated in our program. Patients with lesions accessible to a simple transoral biopsy without endoscopic assistance were not included. Patients unable to tolerate flexible endoscopy or who had a history of bleeding disorder were also ineligible for an in-office procedure. Patients with non-squamous cell carcinoma or a benign diagnosis were also excluded. The final cohort comprised 114 patients (44 in-office biopsy group and 70 operative endoscopy group) (Fig. 1).

### Outcomes

The primary outcome of interest was the time from the date of initial consultation by an otolaryngologist to the date of tissue biopsy. Secondary outcomes were the time from initial consultation to treatment, time from cancer diagnosis to multidisciplinary oncology consultation (MDOC), time from consultation to histopathological diagnosis and time from consultation to treatment.

### Statistical analysis

Categorical variables were analyzed using either the Chi square or Fisher’s exact test as appropriate. Continuous variables were analyzed using Students t-test or a Wilcoxon rank-sum test as appropriate. *P*-values of < 0.05 were considered statistically significant for all tests. Statistical analyses were performed using Stata 14.2 (StataCorp LP, College Station, Texas, USA).

## Results

One hundred fourteen patients were eligible for inclusion in the study (Fig. 1). The operative endoscopy biopsy group had 70 patients while the in-office biopsy group had 44 patients. Demographic information for the study population are summarized in Table 1.

**Table 1:** Demographics of the Study Population

Characteristic	Biopsy Group		*p*-value
	Operative Endoscopy	In-Office	
	*n* = 70	*n* = 44	
Age			**0.04**
Mean years [SD]	60.9 [8.2]	64.3 [8.6]	
Range	37.1–81.3	51.1–86.6	
Gender			ns
Male	64	36	
Female	6	8	
Clinical Stage			ns
In-situ	3	1	
I	4	4	
II	2	3	
III	5	4	
IVA	53	29	
IVB	3	3	
Biopsy Site			ns
Tongue base/vallecula	51	28	
Larynx	16	12	
Pharyngeal wall	3	2	
Other	0	2	
FNA Performed			**< 0.001**
Yes	37	4	
No	33	40	
Treatment			ns
Surgery alone	8	4	
RT alone	7	8	
Surgery and adjuvant RT	4	0	
CRT	48	30	
Surgery and adjuvant CRT	1	1	
Neoadjuvant CRT and surgery	2	1	

*RT* radiation therapy, *CRT* chemoradiation therapy, *ns* non-significant

Bold illustrates a statistically significant result

Patient ages ranged from 37 to 87 years, with a mean age of 62.3 years (SD = 8.4). Patients were predominately male (88%) and presented most commonly with clinical stage IV disease (72%). The tongue base/vallecula were sampled most frequently (69%). Chemoradiation therapy was the commonest modality of treatment (68%). Subjects in the operative endoscopy biopsy group were younger, more likely to have a laryngeal primary and more likely to have fine needle aspiration (FNA) biopsy performed prior to biopsy of the primary cancer. There were no biopsy-related complications in either group.

Table 2 shows the time delays for different events. The mean time delay from consultation to biopsy was 17.4 days for the operative endoscopy biopsy group and 1.3 days for the in-office biopsy group (*p* <  0.0001). The time from consultation to tumor diagnosis was also significantly shorter in the in-office biopsy group (23 vs 7.5 days for the operative endoscopy biopsy and in-office biopsy groups respectively, *p* <  0.0001). There was no difference in the time taken to generate a pathology report between the 2 groups. There were no differences in lag times from initial consultation to MDOC, from MDOC to treatment or from initial consultation to treatment suggesting that access to MDOC and treatment is a challenge regardless of how quickly a histopathological diagnosis is made. Further analysis revealed that MDOCs are triggered by any diagnosis of cancer, including an FNA. Fifty three percent of operative endoscopy biopsy patients had an FNA compared to fewer than 10% of in-office biopsy patients (Table 1). Because FNA was often performed prior to biopsy of the primary lesion, FNA patients entered the queue for MDOC sooner than patients without an FNA, potentially confounding this result.

**Table 2:** Time Delays to Clinical Events

Event	Biopsy Group		*p*-value
	Operative Endoscopy	In-Office	
	*n* = 70	*n* = 44	
ENT Consultation to Biopsy			
Mean days (95% C.I.)	17.4 (13.5–21.3)	1.3 (−0.2–2.9)	**< 0.0001**
ENT Consultation to Diagnosis			
Mean days (95% C.I.)	23.0 (18.8–27.2)	7.5 (5.5–9.4)	**< 0.0001**
Pathology Delay (Biopsy to Diagnosis)			
Mean days (95% C.I.)	5.6 (4.9–6.4)	6.1 (4.9–7.3)	ns
ENT Consultation to MDOC			
Mean days (95% C.I.)	23.4 (19.4–27.4)	19 (16.0–22.0)	ns
MDOC to Treatment			
Mean days (95% C.I.)	33 (27.0–39.0)	32 (28.3–35.7)	ns
ENT Consultation to Treatment			
Mean days (95% C.I.)	51.7 (46.6–56.8)	49.6 (44.6–54.6)	ns

*MDOC* multidisciplinary oncology consultation, *95% C.I.* 95% confidence interval, *ns* non-significant

Bold illustrates a statistically significant result

## Discussion

Diagnosis of suspicious laryngopharyngeal lesions is usually managed by otolaryngologists. Often suspicious lesions can be seen, and accessed, by the transoral route and in those cases a simple transoral biopsy can be performed in the office. However, many patients have lesions that are not accessible transorally and in those cases biopsy in the operating room becomes necessary. The adoption of flexible fiber-optic nasopharyngoscopes has made the assessment, biopsy, and diagnosis of suspicious lesions comparable to that of operative endoscopy biopsy with minimal risk and discomfort [6, 8]. This technique, when used in suitable patients, avoids a general anesthetic, reduces the need for operative resources, and offers the potential for earlier diagnosis and treatment.

In our case series, patients were able to receive an in-office tissue biopsy 16 days earlier and a tumor tissue diagnosis 14.5 days sooner than a biopsy performed in the operating room. The availability of side-channel equipped office endoscopes makes in-office biopsy procedures feasible. Most patients are able to receive a tissue biopsy on the same day as their consultation or within a few days of presentation when side-channel forceps were needed. In addition to an earlier biopsy time, our data illustrate the safety of in-office endoscope-guided tissue sampling, with no procedure-related complications. This finding agrees with studies published elsewhere [6–9].

We were able to obtain tissue sufficient for histopathologic diagnosis in all cases where in-office biopsy was attempted. Analysis of healthcare costs avoided by in-office biopsy was beyond the scope of this study but other authors have reported cost outcomes. In a case series by Naidu et al. (2012), diagnostic in-office biopsy represented a 77% reduction in costs when compared to operative endoscopy biopsy for all patients presenting with laryngopharyngeal tumors [8]. Because all 44 patients in our in-office biopsy group received diagnostic biopsies, a referral to our pre-admission clinic for anesthesiology and internal medicine consultation, and scheduling for operative time and perioperative care were avoided. This likely resulted in an overall reduction in costs to our healthcare system.

Despite the shorter time delay to biopsy and potential for cost savings, some authors express concern regarding the diagnostic clarity of in-office tissue sampling. Richards et al. (2015) reported pathological variability between in-office and operative endoscopy biopsy of laryngeal lesions, with a relatively low sensitivity of 60% [9]. Of particular concern was the identification of invasive squamous cell carcinoma (SCC); just 15% of in-office biopsies proved positive for SCC on initial in-office evaluation. These authors therefore concluded that while in-office biopsies were a safe alternative to operative endoscopy biopsy, they were only moderately successful in identifying dysplastic lesions. Cohen et al. (2013) reported a similarly low sensitivity of 69% and false-negative rate of 33% when compared to direct operative endoscopy biopsy [10]. These values were comparable to results published in a subsequent study of laryngeal biopsies [11]. The authors therefore recommended that suspicious lesions returned as benign pathology or carcinoma in-situ proceed directly to microlaryngoscopy for histopathological verification. While these study results contrast with other published reports [6–8], they nevertheless highlight that variables such as biopsy size, depth, and patient tolerance are potential causes of false negative results. These factors need to be considered in the context of the entire clinical picture when determining patient candidacy for in-office biopsy.

Our study found that the time delays from diagnosis to MDOC, MDOC to treatment, and overall time to treatment were equivalent. This is in contrast to Lippert et al. (2014), in which the authors reported an average time saving of 24.6 days to treatment for patients who received a successful in-office biopsy [6]. In our series the delays from diagnosis to MDOC and treatment were similar between the in-office and operative biopsy groups and the reasons for this are likely multifactorial.

First, patient referrals for MDOC at our cancer center are triggered by a histopathological diagnosis of cancer. Incoming referrals are triaged and reviewed weekly by the Head and Neck Tumor Group. Patients with a diagnosis of cancer on FNA are offered an MDOC even in the absence of a proven primary. Operative endoscopy biopsy can therefore occur after the MDOC and this explains why there were no differences in lag times between the 2 groups.

Second, patients requiring radiation therapy need dental consultation as well as subsequent appointments for fitting of a custom head and neck mold prior to the initiation of radiation. This can take up to 2 weeks, or longer, to complete adding further delay to the initiation of therapy. Third, because 37 of the 70 patients in the operative endoscopy biopsy group initially presented to the MDOC with an FNA biopsy positive for neck malignancy, referral bias is likely present within our population. We therefore believe that a combination of system factors and referral bias explains why we did not see significant reductions in treatment delay in the in-office biopsy group.

Our study has some limitations, primarily its retrospective design and the potential for referral bias as noted above. Despite these shortcomings, the impact on time to biopsy and diagnosis is large and, we believe, real.

Delays in the initiation of treatment for head and neck carcinoma may lead to worse oncologic outcomes therefore strategies that reduce temporal delays ought to be identified and adopted. Our study shows that in-office flexible fiber-optic endoscope-guided biopsy represents a statistically and clinically significant method to expedite the identification of suspicious laryngopharyngeal lesions when compared to operative endoscopy biopsy. However, the overall time to the initiation of treatment, was not significantly impacted by in-office biopsy. We believe further study will clarify these uncertainties and identify opportunities for efficiency. Further study on the economic impact of in-office biopsy will also be helpful in understanding the true benefit of this procedure.

## Conclusions

Our results show that in-office biopsy significantly reduces the time from initial presentation to diagnosis in patients with suspicious laryngopharyngeal lesions presenting to otolaryngologists. The temporal gains via in-office biopsy did not translate into faster access to treatment and we believe that the reasons for this are multifactorial. Further study is needed to quantify the economic impact on healthcare resource utilization.

## Abbreviations

FNA: Fine needle aspiration
MDOC: Multidisciplinary oncology consultation
OR: Operating room
SCC: Squamous cell carcinoma
